# The Cholera, and the Influences Which Favor and Retard Its Spread or Diffusion

**Published:** 1850-03

**Authors:** N. S. Davis

**Affiliations:** Prof. Phys. and Path. in Rush Medical College; Chicago


### ARTICLE VI.

*The Cholera, and the Influences which Favor or Retard its Spread or Diffusion.*—By N. S. DAVIS, M.D., Prof. Phys. and Path. in Rush Medical College.

It is not my present purpose to tax the patience of the reader with any attempt to solve the long vexed question of

Contagion, or Non-Contagion, as applied to Cholera; but to invite his attention to a widely different, and I hope more profitable point of inquiry,—for whether the disease spreads by Meteorological or Contagious causes, it is evident that their action is amenable to the influences of temperature, climate, and various other agencies of a strictly local character. To determine the circumstances under which these influences act, and the extent of their control over the spread of the disease, is a matter of very great importance in a prophylactic or sanitary point of view. In pursuing our task, we shall, as far as possible, consider the influence of each agent separately: and first of Caloric or Temperature:

There is a great tendency among men to draw hasty conclusions, and to use sweeping generalizations, without paying sufficient regard to the particular facts on which they are based. Hence we are often told that the *epidemic* cholera, in its ravages, respects neither country, climate, season, or temperature. Indeed, it is so much easier to express opinions and invent theories, than to patiently observe, and carefully record facts, that we are often surprised at the paucity of authentic items in relation to diseases about which volumes and volumes have been written.

The influence of temperature on the spread of the epidemic cholera, is manifested in two ways. First, by the season of the year when it prevails, and second, by the particular temperature connected with its onset, progress, and decline.

In regard to the influence of seasons, we may observe, that in India, where the Cholera appears to be as indigenous as the Yellow Fever is in some parts of our country, it uniformly makes its appearance during the warm season, and declines at the approach of cold. And if we examine the date on which the disease made its appearance in the principal towns intervening between it [illegible]ome in India and the west of Europe, during the years 1845-[illegible] [illegible]iven in the exceedingly valu- [illegible] history of Dr. Verrollot, we shall find, that of more than seventy-five cities and towns marked on his map, in only three did the disease make its appearance during the winter months, of December, January, and February. Thus, during the year 1845, it had penetrated as far as Bokhara in Nov., where it remained until February 1846, when it appeared at Mechad, but still spreading slowly, it did not reach Asterabad until May. Between this time, however, and the following November, it extends westward to Bagdad, southward to Mecca, and northward to Derbent on the west shore of the Caspian Sea, where it took up its winter quarters and remained until the latter part of March or April, 1847. On the 24th of May it had extended no farther than Kislair, a few leagues north of Derbent; while during the following four months of of June, July, August, and September, it traversed the breadth of the Russian Empire, reaching Valdai, only a few leagues from St. Petersburgh, on the 30th of the latter month. If we follow the disease through the two following years, 1848–9, in its spread along the western borders of Europe, and on this continent, we shall be able to trace the influence of temperature even in a more marked degree than elsewhere.—Hence out of more than two hundred towns and cities, in which the disease has prevailed since 1845, it has commenced its ravages during the warm season of the year, in all except four or five. The only marked exceptions on this continent, are New-York and New-Orleans, in both of which it made its appearance in the month of December, 1848; but under circumstances which strongly coroborate, instead of disprove, the influence of temperature. Thus, from the 2d of December, when the ship New-York landed at the Quarantine Station near New-York City, with cholera on board, to the 22d of the same month, the thermometer ranged from 35° to 68°, with a mean average of about 50° of Fahrenheit. This, although several degrees above the mean temperature of the same time in ordinary years, is still much below a summer heat.—During all this time the disease continued to prevail among the emigrants taken from the ship and other inmates of the quarantine hospitals, and although more than one hundred of the emigrants in direct contact with it, contrived to scale the quarantine walls and escape to the city with more or less of their effects; and though two or three of these were actually seized with the disease and died after they had reached the city, yet the disease exhibited not the slightest disposition to spread beyond the quarantine walls, either on Staten Island or in the city. During the last week in December, and the first in January, the thermometer fell to a mean of only 20°, the extremes being 34° and 9°, and every vestige of cholera disappeared from the quarantine, having occasioned in all 60 deaths. If we contrast these circumstances with those of a similar nature at New-Orleans, where the cholera was introduced during the same month, only nine days later, and in the same manner, viz: by emigrants on board the ship Swanton, which landed on the 11th Dec., we shall have as fair an illustration of the influence of these circumstances as could be obtained by the best devised series of experiments. While the cholera was a whole month in the quarantine and city of New-York, destroying less than sixty lives, wholly refusing to spread with a mean temperature of 50°, and entirely ceasing when it fell to 20°, in New-Orleans it appeared under a temperature almost equal to summer heat, the mercury rising several days during the month of December to 80°, and was in a few days committing its ravages in almost every section of the city, and in less than thirty days some 1,400 victims had found their graves. During the following months of January and February, the temperature fell so much that several frosts occurred, and with them a very decided abatement of the epidemic. Still, the disease lingered in a mild degree until the first of May, when a great amount of rain fell, and the temperature rose to nearly full summer heat, and the bills of mortality showed a corresponding increase in the fatality of the disease. Thus, during the week ending April 28th, there were reported 60 deaths; the following one, ending May 5th, 114; and it continued thus until the 20th, when it began again to decline. Its spread up the river, and throughout the great valley, illustrates, if possible, still more forcibly, the influence of temperature.

Thus, while the epidemic was severe in the city of New-Orleans during the month of December, 1848, thousands of the emigrants and citizens left the city in steamboats up the Mississippi, landing at almost every intermediate place between that city and Cincinnati. On board of a large number of these boats, passengers sickened and died with the disease in its severest forms, during the transit up the river; and some were carried sick with it even so far as Cincinnati, in the months of January and February, where the late Prof. Harrison gave two clinical lectures over well marked cases, in the Commercial Hospital, in that city. Yet in the March number of the Lancet, he tells us that subsequently to his second lecture, "the cholera had entirely disappeared, both in the city generally, and in the wards of the hospital." And so late as the 30th of April, 1849, the editor of the Lancet writes, that "of one thing we are certain, namely: a number of fatal cases, probably from ten to fifteen, have originated in this city within the last two or three weeks; and as they have not sensibly increased for the last ten days, we are led to *hope* that we may be spared a severe visitation," &c. In the May number of the Western Journal, published at Louisville, the editor states that "at New-Orleans it ceases to excite any uneasiness, although deaths from it continue to be reported. No mention of the disease is made in the last Nashville papers. In St. Louis fatal cases were occasionally occurring at the last dates, and boats from the city, as well as from New-Orleans, have brought cholera patients to Louisville within a few days.—In this place, scattering cases are spoken of by our physicians, but there does not appear to be the slightest tendency in the disease to become general."

The truth is, that while the boats from New-Orleans distributed cholera patients during the three months of December, January and February, to almost every village and city in the Valley of the Mississippi and its tributaries, in no place north of Memphis, did it show any disposition to spread or become epidemic until the following spring. So true was this, that so late as March and April, eminent members of the Profession in Cincinnati, St. Louis, Louisville, &c., began to flatter themselves with the hope that those cities would escape any serious visitation of the pestilence. But as the season advanced, all these hopes vanished, and the months of June and July found them all immersed in the pestilence, quite as severe as it had been in New-Orleans.

The foregoing facts show very conclusively that cholera is emphatically a disease of the warm season, and that, with the single exception of some places in Russia, whenever it has prevailed during the winter months, it has been in coincidence with a temperature altogether above what is usual for the corresponding season in other years, as in New-Orleans, and the quarantine of New-York. And I have no doubt but proper investigation would show sufficient local influences, either in the habits or circumstances of the inhabitants, to account for the apparent exceptions in Russia. It is much to be regreted that in so few places where cholera has prevailed, accurate meteorological records have been kept from the be-beginning to the end of its ravages. But so far as these records have been kept and published in a reliable form, they pretty uniformly show that the disease commences at a time when the atmosphere is both unusually moist and warm for the season or locality—it increases with the increase of heat and moisture, until it reaches its acme, and then as regularly declines. When it commences in a place early in the season, before full summer temperature arrives, it spreads slowly, and is a long time in reaching its highest degree of intensity. This is beautifully illustrated by the two seasons of its prevalence the city of New-York. Thus, in 1832, it made its first appearance on the 25th of June, and reached its maximum in the short space of thirty days; while in 1849, it began as early as the 11th of May and spread so slowly that it did not reach its greatest intensity until the latter part of July, a period of at least seventy days. And it is a striking fact in relation to its prevalence in our country, that whatever may have been the period of its appearance in a place, it has never manifested a disposition to spread with rapidity so long as the thermometer remained at mid-day below 75° or 80° Fahrenheit. Thus we are told by Dr. Fenner, that at New-Orleans, al though in the month of December, while the cholera was increasing rapidly, the mercury often rose above 80°—in St. Louis during its rapid increase in the latter part of June the mercury often stood at 90° in the shade. In Louisville. Dr. Bell states that it increased most rapidly from the 25 to the 28th of June, during which time the thermometer ranged from 72° to 76° at sunrise, and often rose to 92°, in the shade, by 3 o'clock P. M. The same coincidence between a high temperature and the commencement of the disease in a truly epidemic form, was noticed in Lexington, Cincinnati, Columbus, Chicago, Buffalo, Albany, Boston, New-York, Philadelphia, and indeed every other city where it has prevailed during the past year in our country. Thus, if we take New-York city as an example, we find that though the disease commenced early in May, with a mean *morning* temperature of only 55°, its mortality up to June 20th, had increased to no more than 144 per week, and the mean temperature to 65°. While from this time on through the months of July and August the mean morning temperature ranged from 70° to 80°, and the disease had increased so rapidly that during the week ending on the 21st of July, the number of deaths was 714, or more than 100 per day; and on the 6th of October, when the mean temperature had again fallen to 55°, the epidemic had effectually ceased. It must be borne in mind that the temperature here given is that of the morning, the maximum during mid-day generally ranging from 10° to 15° higher. Concerning our own country, I think no further evidence need be adduced to satisfy every candid mind, that temperature exerts a very important, if not a controlling, influence over the production and spread of epidemic cholera.

The next agent which will claim our attention, is *humidity*, or moisture.

To show the effects of this agent in favoring the onset of the cholera, we need not go beyond our own country, or refer to its prevalence anterior to the year 1848. Thus, Dr. A. Hester, of New-Orleans, referring to the month of December, states that "for some days prior to the appearance of the first case of cholera, (in that city) the rain fell almost daily,—the atmosphere was *humid*, *murky*, *close and oppressive*." At Columbus, Ohio, says the late editor of the Ohio Med. Journal, "the first unequivocal case occurred on the 21st of June, '49." From that time to the 1st of July, "slight showers of rain fell almost every day," rendering the atmosphere so moist as to show by actual experiment, "*a perfect saturation*." At Nashville, says Dr. H. B. Walton, "the weather for the greater part of the time, has been warm and *wet*." In Clarksville, according to Dr. E. B. Haskins, "the weather, for several weeks prior to the invasion of the epidemic, had been *cloudy and rainy*, with sudden alternations of temperature. The atmosphere, for most of the time, was hazy." The epidemic commenced in New-York about the 11th of May, in one of the lowest and dampest portions of the city. We are told by Dr. Wm. P. Buel, Physician to the Centre Street and Thirty-fifth Street Hospitals, that "the month of May *was wet and damp*, the prevailing wind N. E., and 3.77 inches of rain fell." While it prevailed in the quarantine hospital during the December previous, Dr. J. W. Sterling, one of the Physicians in that establishment, says, "the weather, since the arrival of the packet ship New-York, has been *wet* and quite uncomfortable, though so mild until the 22d of December, that the furnaces of the lower hospital kept the wards at a comfortable temperature." The actual amount of rain that fell during the month, was 6.30 inches.

We have been unable to find any reliable meteorological record, corresponding with the onset of the disease, either in St. Louis, Cincinnati, or Buffalo; but it became manifestly epidemic and severe in these cities about the last of May, a period characterized by great moisture in the atmosphere in all those places where records were kept, as we have already seen, at Columbus, Ohio; at New-York, and at Nashville, Clarksville, Memphis, and New-Orleans, on the Mississippi. In New-Orleans, the amount of rain that fell during the month of May, was 12.855 inches; in Charleston, S. C., 3.80 inches; in New-York, 3.77. In reference to Chicago, Prof. Evans says that "at the time of the great increase in the mortality, about the 20th of July, the weather, for a few days only, was exceedingly warm and sultry;" and we find by a reference to the table which he has published, that 17 out of the 30 days following the 20th of July, were damp or rainy; making up nearly the whole period through which the disease prevailed in this city. The influence of humidity is still further shown by the particular locations most frequently attacked. On this point, we shall find, on close examination, a complete coincidence between the facts in this country, and those of Europe, as announced in the report of the Sanitary Commissioners of London in 1848. The commissioners assert, "that the mode of invasion of cholera in the various cities of Europe, has been every where strikingly uniform. It has almost always made its first outbreak in the *lowest* and *dampest* portion of the city attacked. They verify this statement by references to St. Petersburgh, Dantzic, Berlin, Moscow, Breslau, Warsaw, Paris, Sunderland, Carlisle, Manchester, London, and England generally. "And," says the able writer in the British and Foreign Medico-Chirurgical Review, "this conclusion is borne out by the evidence of the best writers on cholera, particularly by Orton and Jameson." In reference to Buffalo, Dr. A. Flint states in the Buffalo Medical Journal, that "the sections of the city in which the epidemic prevailed *most*, and with the *greatest virulence*, were, first, streets in close proximity to the canal, densely inhabited, and abounding most in poverty and vice; second, a part of the city known as the Hydraulics, in which miasmatic affections are somewhat rife, and the population, generally speaking, not of a character to be peculiarly exempt from an epidemic disease; and third, in a part lying in a North-Eastern direction from the city, in which the population consists almost wholly of German laborers."

Having already shown that when the disease spread to most places on the Mississippi and its tributaries, the season was wet and rainy, we shall not attempt to investigate the influence of particular locations in them. Indeed, so loose, general, and sometimes contradictory, are the statements of different writers in regard to all such topics, that it is impossible to get reliable data or facts concerning any place, without a *personal* knowledge of its location, and all the local circumstances connected with its several parts. And this will continue to be the case, until those having charge of the sanitary condition of towns and cities, take the necessary measures for ascertaining and recording the prevalence and progress of epidemic and endemic diseases, with the coincident condition of the air, the soil, the habits of the people, &c., with proper maps and tables embodying the whole in an authentic and perfectly reliable form. If this was done, we should have all the requisite elements for comparison, and we should soon be able to separate the mere accidental or coincident circumstances, from those which are constant or essential. Yet inestimably valuable as such knowledge would be to the human family, it seems almost impossible to induce the proper authorities to make any adequate provision for obtaining it.

To ascertain the *number* of victims who have fallen, and daily herald it to the public in the manner best calculated to frighten the living, and to abate some of the most unimportant nuisonces, seems to be all that our Boards of Health and Sanitary Committees deem necessary. Hence, if we search ever so carefully through the numerous Medical Periodicals of the past year, we shall find *bills* of *mortality*, to constitute almost the only important item that is left us concerning the epidemic that has just made its second sweep over our country. And with only two or three partial exceptions, we shall look in vain for a full and satisfactory history of its prevalence in any single city on the continent.

Among the particular exceptions to which we allude, is the account of the cholera in the city of New-York, by Wm. P. Buel, M.D., in the Jan. No. of the New-York Journal of Medicine, which, though very deficient in meteorological, geological and geographical facts, is nevertheless accompanied by a map, which, to those personally acquainted with the whole ground occupied by the city, is exceedingly valuable. And nothing demonstrates more clearly the important influence of local circumstances, than the facts connected with the prevalence of cholera in that city, especially when we compare the past year with the summer of 1832. It should be remembered that the city occupies a triangular space, the two sides of which are washed by the Hudson and East Rivers, while its base is stretched across the central unoccupied part of the Island. The ground was naturally uneven, giving rise to several low, marshy places, which were more or less perfectly filled up as the city extended its borders. Some of these places, however densely populated, still remain much depressed below the general level of the city. One of the most marked of these is a tract beginning at the City Prison on Centre Street, extending through the *Five Points*, across Chatham Street in the neighborhood of James and Rosevelt Streets to the East River; and thence North-eastward in the direction of Cherry Street to Corlear's Hook. The same may be said of that part of the 13th and 11th Wards bordering on the East River and extending between Grand and 14th Streets; and that part of the 1st Ward, occupied by the South end of West, Washington, and Greenwich Streets, bordering on the Hudson River. Naturally there existed another low, wet tract from the junction of Canal Street and Broadway in a West and North-western direction, through the 8th and part of the 9th Ward.

Now if the reader will bear these geographical facts in mind, while he casts his eye over the map given by Dr. D. M. Reese, in his account of the cholera in New-York during the summer of 1832, he will at once perceive their important bearing; for it was precisely in these sections that the disease spent its principal force and violence during that season, as abundantly shown by the detailed reports of the board of health, and the hospital physicians of that year. With the exception of that section mentioned as extending through the 8th and 9th Wards in the direction of Canal, Laurens, and Carmine Streets, the parts of the city before pointed out, have remained with no essential change, except a supply of Croton water. The streets in the 8th and 9th Wards, besides being supplied with Croton water, have, since 1832, been vastly improved by buildings, sewers, paving, &c., &c., so that they rank favorably in their local circumstances, with most of the other wards of the city we compare the map of Dr. Buel representing the prevalence of cholera in 1849, with that of Dr. Reese already referred to, we shall see the result; for while we find the disease again commencing, as in 1832, in the most sunken part of that tract extending from Centre Street to East River, and extending Eastward and Northward along the borders of that river through the 7th, 13th and 11th Wards; and in the lower part of Washington and West Streets, we find it in the 8th and 9th Wards comparatively light.

Thus, according to the statistics of Dr. Buel, out of the 5.000 deaths from cholera in the whole city, 2,400, or nearly one half, were furnished by the 4th, 6th, 7th, 13th and 11th Wards, being the tract extending from Centre Street through the Five Points to the East River, and then North-eastward along the border of that River to the neighborhood of 14th Street. Of the remainder, 300 occcurred in the 1st Ward bordering on the Hudson, and 778 in the 16th Ward, also bordering on the Hudson River, but in the extreme North-western part of the city,—leaving less than 1,500 for all the other Wards, viz: the 2d, 3d, 5th, 8th 9th, 10th, 14th, 15th, 17th, 18th, and 12th, making up the whole central part of the city, and including at least three fourths of the population. It will be observed that a heavy mortality occurred in the 16th Ward,---the greatest, indeed, except the 6th, of any Ward in the city. And when the reader is informed that this Ward mostly occupies *very high* ground—that it is neither thickly covered with buildings, nor densely populated, he will at once begin to conclude, as others have before him, that it militates strongly against the idea that lowness and dampness favor the prevalence of the cholera. And perhaps no more striking illustration of the necessity of a full and minute knowledge of all the facts, and the danger ot judging from a few, could be adduced than this.

We are so accustomed to associate the idea of dryness with elevation, that we very often judge of the former solely from a knowledge of the latter; when, in truth, mere elevation is neither an index of the relative dryness of either atmosphere or soil, as is shown by the highest part of the 16th Ward, to which we have alluded. The surface of this tract, though mostly elevated, is uneven, and pretty closely underlaid with micaceous rock, which in some places crops out entirely on the surface. The streets are new, and neither supplied to any considerable extent with Croton water, nor drained by sewers. The closely underlying rock, especially in the highest part of the ward, prevents the water that falls on the surface from running off, and consequently every depression on the surface, many of which were formed by filling up new streets across low places, were filled with standing water during the whole spring, until *evaporated* by the heat of summer. And not only so, but during the spring, on the height of the ground, all the new cellars, (and several long rows of new buildings were in the process of erection,) and many of the old ones were filled with water, and after remaining so for weeks, were only emptied by pumping it out. Such was the character of the 16th Ward, so far as regards humidity, although represented as the highest, dryest and most airy portion of the city. I speak of it with minuteness, because it was my fortune to travel over it daily during the spring and summer of 1849, and to attend some of my worst cases of cholera on its highest points. The New York Institution for the Blind is located on this height of ground, concerning which, Dr. Buel speaks as follows, viz: "This institution suffered most severely. It has an open, airy, elevated position, surrounded by spacious streets and avenues, but it forms a portion of the 16th ward, where from the action of local or endemic causes, the epidemic influence operated with intense energy. With about 120 inmates, there were 44 cases of cholera, of which 9 or 10 were fatal, and this with all possible prophylactic measures. We are informed by Dr. Bliss, who had the medical charge of the institution, that but for the timely precaution of removing all the inmates into the country, he believed the greater portion of them would have perished."

We should also add, that in the lowest part of this ward, bordering closely on the Hudson River, were several soap-making, bone-boiling, and other like establishments, and one very extensive distillery, in the stables attached to which, were congregated several hundred cows.

Closely connected with the subject of humidity, is the nature of the soil, and the quality of the water. That these exerted an influence on the progress of the disease in this country, is too manifest to admit of doubt. If our readers will take a map of the Union, and compare the course of the disease, both in 1832 and 1848–9, throughout the whole territory of New-England, together with that extensive range of highlands beginning in the North-eastern part of New-York, and extending along the Catskill and Alleghany ridges to near the Gulf of Mexico, with its prevalence and diffusion over that vast territory bordering on the Mississippi and its tributaries, the St. Lawrence, and that great chain of inland seas or lakes which lie along our Northern boundaries, they cannot fail to observe a manifest difference. It is well known that throughout the whole of the first regions named, the appearance of the disease in a country village was very rare, while its prevalence in a purely farming district was wholly unknown.—While, during the spring and summer of 1849 especially, comparatively few villages, however small, located in the Great Western or Mississippi Valley escaped, and the disease was even more fatal on many of the plantations along the lower Mississippi, than in the most densely populated cities on the Atlantic coast. Not only was the disease more rapidly and extensively diffused in one region than in the other, but it was far more severe and fatal. This is strikingly exhibited by the following table, viz:

| | Population. | Deaths in 1849. | Ratio. |
|---|---|---|---|
| Boston, - - - - - | 120,000 | 602 | 1 in 200 |
| New-York, - - - - | 400,000 | 5,122 | 1 in 79 |
| Philadelphia, - - - - | 300,000 | 1,022 | 1 in 300 |
| Buffalo, - - - - - | 40,000 | 858 | 1 in 46 |
| Chicago, - - - - | 25,000 | 678 | 1 in 36 |
| Cincinnati, - - - - | 100,000 | 4,450 | 1 in 23 |
| St. Louis. - - - - | 90,000 | 4,297 | 1 in 21 |
| New-Orleans, - - - | 150,000 | 4,000 | 1 in 37 |

To what, is this marked contrast in the relative fatality of the disease between the three Eastern and the five Western cities owing? Certainly not to any increased facilities enjoyed by the Western cities for intercommunication of one part of a city with another, or of the city with the surrounding country; neither can it arise from any peculiar character or habit of the people. For, of all the cities on this continent, New York stands foremost as the grand focus and receptacle of the poverty and filth of Europe; and contains by far the largest population of that character which has ever served as the food of epidemics and endemics of any city in our country. And yet her ratio of mortality from cholera during the past year is less than one half of that of the most favored city in the Mississippi Valley, and a little more than one half of that at Buffalo, located on the great internal lakes.

Let us take this marked difference in fatality in connection with the difference of diffusion through country places, mentioned already and ask again why these important variations between the prevalence and fatality of the cholera on the comparatively primitive Eastern sections, and the great tertiary or alluvial soil of the West? Is there any other rational answer except that which refers to the soil, or the geological formation of the two sections? I think not. It was my original design to extend these inquiries concerning temperature, humidity, soil, &c., together with the influence of personal habits, far more minutely, with the sole object of arriving at some valuable conclusions irrespective of all theories; but I find myself already occupying too much space, and my field of investigation only just entered upon. Hence I shall refrain from attempting to deduce any formal conclusions until I resume the subject at a future day.

*Chicago, Feb.* 14, 1850.

